# The Potential for the Internet and Telehealth in Caregiver Support. Comment on “Using Technology to Facilitate Fidelity Assessments: The Tele-STAR Caregiver Intervention”

**DOI:** 10.2196/14953

**Published:** 2021-02-16

**Authors:** Kohei Kajiwara, Jun Kako, Hiroko Noto, Yasufumi Oosono, Masamitsu Kobayashi

**Affiliations:** 1 Japanese Red Cross Kyushu International College of Nursing Munakata Japan; 2 College of Nursing Art and Science University of Hyogo Akashi Japan; 3 Department of Health Sciences Graduate School of Medical Sciences Kyushu University Fukuoka Japan; 4 Faculty of Nursing National Defense Medical College Tokorozawa Japan

**Keywords:** dementia, caregiver, technology

We read with interest the recent paper entitled “Using Technology to Facilitate Fidelity Assessments: The Tele-STAR Caregiver Intervention” by Lindauer et al [1]. The authors concluded that Tele-STAR contributed to low caregiver burden and showed good fidelity as an intervention method.

Internet-based videoconferencing technology is an important source of support for caregivers of persons with dementia. Researchers have previously demonstrated the positive potential of computer-mediated interventions and technology-based cognitive behavioral therapy interventions for caregivers of people with dementia [2,3]. Others have raised the difficulties in measuring intervention fidelity in a consistent manner [4,5], which raises the importance of consistency when considering fidelity evaluations. Moreover, as the study reported results that may be attributed to both in-home and telehealth intervention experiences of participants, it may be useful to consider the interplay of these aspects.

Lindauer and colleagues [1] reported a slight reduction in caregiver burden, attributed to an improvement in caregivers’ responses to patients with dementia, facilitated by the Tele-STAR intervention. Caregiver burden is an important consideration in the field of dementia care. A recent study found an internet-based intervention to be effective in increasing the positive aspect of subjective appraisal for caregivers of persons with dementia [6]. In addition, we have studied the subjective appraisal of both negative and positive aspects in this population [7]. Assessments that take into account both sides of subjective appraisal are capable of providing a broad understanding of a caregiver’s context, and we would argue that an outcome that takes both into account would be more useful than current practices allow for.

The support that can be offered to caregivers using internet-driven technologies should continue to be explored, and the study conducted by Lindauer and colleagues [1] provides useful data in this regard. We agree that internet-based interventions will be beneficial to caregivers of persons with dementia in the future.

## References

[1] Lindauer A, McKenzie G, LaFazia D, McNeill L, Mincks K, Spoden N, Myers M, Mattek N, Teri LL (2019). Using Technology to Facilitate Fidelity Assessments: The Tele-STAR Caregiver Intervention. J Med Internet Res.

[2] McKechnie Vicky, Barker Chris, Stott Josh (2014). Effectiveness of computer-mediated interventions for informal carers of people with dementia-a systematic review. Int Psychogeriatr.

[3] Scott JL, Dawkins S, Quinn MG, Sanderson K, Elliott KE, Stirling C, Schüz Ben, Robinson A (2016). Caring for the carer: a systematic review of pure technology-based cognitive behavioral therapy (TB-CBT) interventions for dementia carers. Aging Ment Health.

[4] Godwin Kyler M, Mills Whitney L, Anderson Jane A, Kunik Mark E (2013). Technology-driven interventions for caregivers of persons with dementia: a systematic review. Am J Alzheimers Dis Other Demen.

[5] Nguyen Tuan Anh, Nguyen Huong, Pham Thang, Nguyen Trong Hung, Hinton Ladson (2018). A cluster randomized controlled trial to test the feasibility and preliminary effectiveness of a family dementia caregiver intervention in Vietnam: The REACH VN study protocol. Medicine (Baltimore).

[6] Moskowitz Judith T, Cheung Elaine O, Snowberg Karin E, Verstaen Alice, Merrilees Jennifer, Salsman John M, Dowling Glenna A (2019). Randomized controlled trial of a facilitated online positive emotion regulation intervention for dementia caregivers. Health Psychol.

[7] Kajiwara Kohei, Noto Hiroko, Yamanaka Makoto (2018). Changes in caregiving appraisal among family caregivers of persons with dementia: A longitudinal study over 12 months. Psychogeriatrics.

